# Can Wound Exudate from Venous Leg Ulcers Measure Wound Pain Status?: A Pilot Study

**DOI:** 10.1371/journal.pone.0167478

**Published:** 2016-12-09

**Authors:** Taichi Goto, Nao Tamai, Gojiro Nakagami, Aya Kitamura, Ayumi Naito, Masayuki Hirokawa, Chisako Shimokawa, Kazuo Takahashi, Junichi Umemoto, Hiromi Sanada

**Affiliations:** 1 Department of Gerontological Nursing/Wound Care Management, Graduate School of Medicine, The University of Tokyo, Bunkyo-ku, Tokyo, Japan; 2 Global Leadership Initiative for an Age-Friendly Society, The University of Tokyo, Bunkyo-ku, Tokyo, Japan; 3 Japan Society for the Promotion of Science, Chiyoda-ku, Tokyo, Japan; 4 Fujisawa City Hospital, Fujisawa-city, Kanagawa, Japan; 5 Ochanomizu Vascular & Vein Clinic, Chiyoda-ku, Tokyo, Japan; Tokai Daigaku, JAPAN

## Abstract

We investigated the associations between the self-evaluated pain status and two pain biomarker candidates, nerve growth factor and S100A8/A9, in exudate from venous leg ulcer to finally develop an objective pain evaluation method. Patients with venous leg ulcer participated in this cross-sectional observational study conducted between April and October 2014 at two medical facilities. During routine wound care, each participant self-evaluated their pain status at each examination using the 10-point numerical rating scale (present pain intensity) and the short-form McGill Pain Questionnaire 2 (continuous pain, intermittent pain, neuropathic pain, affective descriptors, and total score). Venous leg ulcer exudate sample was collected after wound cleansing. The nerve growth factor and S100A8/A9 concentrations in the venous leg ulcer exudate were measured by enzyme-linked immunosorbent assay and standardized according to the wound area. The association between each pain status and the two standardized protein concentrations was evaluated using Spearman’s correlation coefficient. In 30 sample collected from 13 participants, the standardized nerve growth factor concentration was negatively correlated with continuous pain (*ρ* = -0.47, *P* = 0.01), intermittent pain (*ρ* = -0.48, *P* = 0.01), neuropathic pain (*ρ* = -0.51, *P* = 0.01), and total score (*ρ* = -0.46, *P* = 0.01). The standardized S100A8/A9 concentration was positively correlated with present pain intensity (*ρ* = 0.46, *P* = 0.03) and continuous pain (*ρ* = 0.48, *P* = 0.03). Thus, these two proteins may be useful for objective evaluation of wound pain in venous leg ulcer patients.

## Introduction

Venous leg ulcer (VLU) is one of the most common chronic wounds. The prevalence of VLU is 1% in the adult population in western countries, and its incidence increases with age [1, 2]. Remarkably, approximately 80% of VLU patients experience various types of severe pain including nociceptive and neuropathic pain [3–5]. Wound pain is related to wound healing and decreases the quality of life in patients [6, 7]; as a result, prevention of pain during wound care has been emphasized [8]. To provide appropriate pain management, we must accurately and quickly assess the patient’s pain status including both the intensity and quality.

Although several subjective pain evaluation tools such as the visual analog scale, numerical rating scale, and the McGill Pain Questionnaire are available, they may not be usable in patients with cognitive impairment because they require voluntary participation of the patient to be used accurately [9]. Furthermore, an instrument, PainVision^™^ system which can measure pain intensity more quantitatively has been tried to use for cognitively intact patients with wounds but it is also difficult for cognitively impaired patients to understand how to use it [10]. An objective pain evaluation is greatly needed in the current super-aged society in which the prevalence of dementia is significantly increasing.

Several studies have sought a tool to objectively evaluate pain. The Pain Assessment in Advanced Dementia and the Abbey Pain Scale which evaluate patient’s facial expression, behavioral change, physiological change and so on are promising objective pain evaluation scales for cognitively impaired patients and are used clinically [11, 12]. These scales were not designed for assessment of wound pain; thus, they do not assess whether pain is caused by a wound or other pathology. For wound pain, changes in skin conductance have been studied as a method to evaluate surgical wound pain [13], but this biological reaction also do not assess the source of pain and can be affected by any stressor in addition to pain.

To address these challenges, we have investigated potential biomarkers associated with pain generation as markers of wound pain. We previously reported that a high temperature specifically at the periwound area on thermography may well be an indicator of a painful wound [14]. However, thermography cannot be used to assess neuropathic pain that is not accompanied by a temperature elevation. Therefore, wound pain biomarkers that can detect both types of pain are needed.

In the present study, we examined the usefulness of pain biomarkers in wound exudate. We previously demonstrated that wound exudate may reflect the changing wound status and can be collected non-invasively [15–17]. For selecting pain biomarker candidates, we focused on the pathology of VLU. Leukocytes including neutrophils accumulated around wound area cause inflammatory reaction, resulting in producing inflammation-related proteins. One of the important proteins is nerve growth factor (NGF) because it is associated with increasing number of nociceptors and pain-related neurons among not only myelinated (Aδ) fibers but also unmyelinated (C) fibers, resulting in hypersensitivity [18]. Additionally, it activates neutrophils [2]. Furthermore, the neutrophils also produce S100A8/A9, which is associated to prostaglandin E_2_ (PGE_2_) production [19, 20]. NGF and PGE_2_ are well known as algesic substances and related to neuropathic pain generation [21, 22]. Among these molecules, NGF and S100A8/A9 were offered to this study as pain biomarkers because of stability of protein compared with other chemical agents. In order to determine whether measurement of NGF and S100A8/A9 protein concentrations could be used to assess pain, we examined the association between pain biomarkers isolated from VLU exudate and the self-evaluated pain intensity and quality by patients without cognitive impairment.

## Materials and Methods

### Study setting and participants

A cross-sectional observational study was conducted at two medical facilities: a general hospital in an urban area of Kanagawa, Japan and a private vascular surgery clinic in an urban area of Tokyo, Japan. Participants were recruited and observed between April and October 2014. Patients aged over 20 years and diagnosed with VLU who could accurately use subjective pain evaluation scales were included in this study. Patients who refused to participate this study, or were cognitively impaired or in critical condition determined by the physician were excluded. Although this study was cross-sectional, the observational procedure was repeated at all of the hospital visits of each participant.

### Observation protocol

The protocol was approved by the Ethical Committees of the Graduate School of Medicine of the University of Tokyo (#10389–2), and the two medical facilities. Written informed consent was obtained from each enrolled patient.

After the physician or nurse recruited eligible patients, two researchers observed several parameters during the participants’ routine wound care. Basically, trained one investigator (T.G.) always collected data mainly and other two investigators (A.K. and A.N.) supported it based on a protocol to assure the data quality and to minimize technical bias. Demographic data including age, sex, body mass index (BMI), and wound age were obtained from medical records or through interviews with the participants. Initially, the present pain intensity was evaluated using a 10-point numerical rating scale (NRS) of which the range was 0–10; higher was worse condition. After cleansing the wound, digital photographs of the wound were taken, and the exudate was collected by the researchers. After receiving such data collection, the participants completed the Japanese version of the short-form McGill Pain Questionnaire 2 (SF-MPQ-2) [23–25] to evaluate wound pain intensity for each type of pain quality. SF-MPQ-2 asks pain intensity of a past week and is consisted by four subscales: continuous pain (range 0–60), intermittent pain (0–60), neuropathic pain (0–60) and affective descriptors (0–40); a higher score is worse condition. Cronbach’s alpha of SF-MPQ-2 was 0.95 in the present study. The wound status was evaluated by the DESIGN tool [26] by the researchers. DESIGN tool is consisted by six components: depth, exudate, size, inflammation/infection, granulation tissue, and necrotic tissue. Each item is scored in three to seven grades; a higher score is worse condition. A researcher (T.G.) blindly measured the wound area on the digital photograph three times by tracing the wound edge using ImageJ software program (National Institutes of Health, MD, USA), and the mean area was subjected to analysis. The percent coefficient of variation for wound area measurement was 3.4% in the present study. When a participant had plural wounds, the most painful wound was observed.

### Exudate collection

After cleansing the wound, two sheets of sterile gauze (Hakujuji Co., Ltd., Tokyo, Japan) were placed on the wound. The gauze was removed 3 minutes later and soaked in 10 mL phosphate-buffered saline in a 50 mL centrifuge tube (Corning Japan, Tokyo, Japan). The collected exudate samples were stored at -80°C until analysis. We confirmed that presence of any protein was collected for each sample by measuring the total protein concentration using a Micro BCA^™^ Protein Assay Kit (Thermo Fisher Scientific Inc., IL, USA).

### Enzyme-linked immunosorbent assay (ELISA)

NGF concentrations were measured using a Human Beta-NGF ELISA Kit (RayBiotech Inc., GA, USA). S100A8/A9 concentrations were measured using a Human S100 Calcium-binding Protein A8/A9 Complex (S100A8/A9) ELISA Kit (Cusabio Biotech, Wuhan, China). All assays were performed according to the manufacturer’s instructions. The frozen exudate samples were thawed and then centrifuged at 15,000 rpm for 30 minutes at 4°C, and the supernatant was used for the analyses. Each measurement was performed in duplicate. In the present study, the percent coefficients of variation for NGF and S100A8/A9 were 2.5% and 9.0%, respectively.

### Statistical analysis

According to the results of Shapiro-Wilk test for testing normal distribution, wound area, concentrations of S100A8/A9, intermittent pain, neuropathic pain, affective descriptors, and total score of SF-MPQ-2 were not normally distributed (S1 Table). Thus, nonparametric analyses were used in the present study. Data are presented as the median with interquartile range (IQR) or the number with percentage (%). When the biomarker concentration was not detected, the sample was excluded from the analysis. The measured protein concentration was standardized according to the wound area. In order to identify usability of proteins in exudate for pain assessment, the strength of the association between the pain intensity and the standardized protein concentration was evaluated using Spearman’s correlation coefficient. The various patient characteristics, especially age, sex, and wound age, were suppose to affect the associations between pain status and protein concentrations warrants examination; thus, we performed stratified analyses for the three factors as supporting data. Classifying all the data into two groups: less or over median age, sex, and less or over 9-week wound age, we performed the analyses if we found statistically significant differences of standardized NGF and S100A8/A9 concentrations and pain statuses between the two groups. Additionally, intra-individual correlations had to be considered in the present study because the participants were repeatedly investigated. However, an analysis that adjusted the correlation could not be performed due to also limited sample size. Alternatively, we examined the change in pain intensity and the standardized protein concentration, and calculated the correlation coefficients in a participant with an ID of “G,” whom we observed most frequently. Additionally, we calculated the correlation coefficients between the pain intensity and the standardized protein concentrations in all samples except those with the ID “G.” A *P*-value less than 0.05 was regarded as statistically significant. Statistical analyses were performed using JMP Pro Version 11.0.0 (SAS Institute Inc., NC, USA) and Stata/IC 14.0 (StataCorp LP, TX, USA).

## Results

### Participant and wound characteristics

The eligibility of 20 VLU patients was assessed; of these, three were excluded due to refusal to participate and thus 17 patients participated to this study. However, three of 17 participants were excluded from the analyses due to wound closure at the first examination. There was no patient who was cognitively impaired or in critical condition of systemic and local diseases such as diabetes, hypertension, and cardiovascular diseases determined by the physician. Additionally, one patient on steroids had to be excluded because steroids can easily affect both the pain status and composition of the exudate.

Seven of the remaining 13 participants were male (53.8%). The median age was 73.0 (47.5–76.5) years, and the median BMI was 26.4 (21.0–33.1) kg/m^2^. The median wound age was 13.5 (5.0–42.0) weeks (Table 1). Of 32 examinations in the 13 participants, two examinations were excluded because the exudate sampling did not follow the protocol and a large amount of local anesthetic was used to perform endovenous laser ablation.

**Table 1:** Participant characteristics (n = 13).

ID	Age	Sex	BMI	Wound age
A	53	Female	25.3	2
B	67	Female	32.5	16
C	65	Male	20.1	UN
D	75	Female	39.3	24
E	42	Male	17.9	24
F	42	Male	26.4	3
G	79	Male	21.9	81
H	73	Male	22.1	11
I	76	Male	32.9	5
J	84	Female	37.8	8
K	77	Female	19.1	67
L	73	Female	33.3	5
M	34	Male	26.7	48
Median (IQR)	73.0 (47.5–76.5)	7 (53.8)^a^	26.4 (21.0–33.1)	13.5 (5.0–42.0)

^a^ Number of male (%). The units of age, BMI, and wound age are years, kg/m^2^, and weeks, respectively. BMI, body mass index; UN, unknown; IQR, interquartile range.

The results of 30 examinations were included in the analysis. According to the wound status as assessed by DESIGN tool, the wound depth of 29 out of 30 examinations was 2-point, defined as a “lesion extends into dermis,” and the volume of exudate of 18 examinations was 2-point and 11 examinations was 3-point, defined as a wound that “requires daily dressing change” and “requires dressing change more than twice a day,” respectively. Moreover, the state of inflammation/infection of 27 examinations was 1-point, defined as a “signs of inflammation.” In 9 examinations, the patient was administered non-steroidal anti-inflammatory drugs (NSAIDs) within six hours prior; NSAIDs use was unknown in five instances (Table 2).

**Table 2:** Wound and pain characteristics during each examination.

ID	Wound area	DESIGN						NRS	SF-MPQ-2					NSAIDs use	NGF	S100 A8/A9
		D	E	S	I	G	N	PPI	CP	IP	NP	AD	TS			
A-0	0.35	2	1	1	1	0	2	7	42	45	37	27	151	UN	4.23	0.25
B-0	5.68	2	3	2	1	1	1	2	21	13	18	8	60	UN	5.70	0.24
C-0	3.19	2	3	2	1	3	1	3	17	10	14	8	49	UN	3.13	ND
C-7	2.97	2	2	2	1	3	0	6	23	11	10	6	50	-	ND	0.30
C-11	4.61	2	3	2	1	3	0	8	42	32	32	11	117	-	3.00	ND
C-18	3.84	2	2	2	1	3	0	8	44	32	21	14	111	-	2.58	3.85
C-22	4.63	2	2	2	1	2	0	9	51	51	49	29	180	-	0.06	ND
D-0	2.10	2	2	1	1	1	0	2	0	0	5	0	5	UN	4.23	0.06
E-2	1.93	2	2	1	1	0	1	0	30	25	22	11	88	UN	1.53	0.07
F-0	0.33	2	3	1	1	0	1	7	22	27	13	12	74	+	1.84	0.21
G-0	12.71	2	2	3	2	4	1	5	30	21	17	6	74	+	2.15	0.58
G-3	13.50	2	3	3	1	2	1	9	31	8	22	8	69	+	2.58	0.91
G-4	16.27	2	3	3	1	2	1	3	19	13	17	0	49	+	7.17	0.64
G-5	16.57	2	2	3	1	2	1	5	23	31	24	0	78	+	4.48	1.79
G-7	15.26	2	3	3	1	1	1	4	26	19	8	10	63	+	4.84	0.79
G-8	13.92	2	3	3	1	2	1	2	7	0	8	0	15	-	ND	0.17
G-10	12.27	2	2	2	1	1	1	5	8	2	8	0	18	+	10.30	ND
G-12	3.87	2	2	2	1	1	1	2	1	0	2	0	3	-	6.07	ND
G-16	2.25	2	2	1	1	1	1	1	1	0	0	0	1	-	3.86	ND
G-22	0.85	2	2	1	1	0	0	0	0	0	0	0	0	-	0.74	0.04
H-0	8.36	2	2	2	1	1	1	2	14	1	2	0	17	-	3.99	0.33
I-0	4.59	2	2	1	1	2	1	5	18	6	3	0	27	-	4.60	0.41
I-4	0.42	2	2	1	0	1	0	0	0	0	0	0	0	+	9.93	0.07
J-0	8.86	3	3	2	1	4	1	0	10	30	18	0	58	-	3.07	0.40
J-2	12.68	2	3	2	1	3	1	2	10	35	25	10	80	-	0.49	0.05
J-5	4.74	2	2	2	1	1	0	0	5	0	3	0	8	-	8.28	0.13
K-0	6.60	2	3	2	2	1	0	0	23	12	23	0	58	-	0.25	0.79
K-3	3.20	2	2	2	1	1	0	3	31	28	36	0	95	-	1.72	ND
L-0	3.01	2	2	1	1	3	1	0	2	0	3	0	5	-	5.03	0.26
M-0	0.43	2	2	1	1	1	1	3	13	9	10	6	38	+	4.84	ND

ID indicates each participant’s ID followed by the follow-up duration from the first examination. NSAIDs use is defined as receiving an NSAID within 6 hours prior to each examination, where (-) = none, (+) = receiving NSAID, and UN = unknown. The first observation of ID “E” and the last observation of ID “H” were excluded. The units for wound area, NGF concentration, and S100A8/A9 concentration are cm^2^, pg/mL and ng/mL, respectively. DESIGN tool is consisted by six components: D, depth (0–5); E, exudate (0–3); S, size (0–6); I, inflammation/infection (0–3), G, granulation tissue (0–5), and N, necrotic tissue (0–2). Each item is scored in three to seven grades; a higher score is worse condition. The range of NRS is 0–10; higher was worse condition. SF-MPQ-2 is consisted by four subscales: continuous pain (range 0–60), intermittent pain (0–60), neuropathic pain (0–60) and affective descriptors (0–40); a higher score is worse condition. NRS, 10-point numerical rating scale; PPI, present pain intensity; SF-MPQ-2, short-form McGill Pain Questionnaire 2; CP, continuous pain; IP, intermittent pain; NP, neuropathic pain; AD, affective descriptors; TS, total score; UN, unknown; NGF, nerve growth factor; ND, not detected.

### Pain status

Table 3 summarizes the self-reported pain status. The median score of present pain intensity assessed using the NRS was 3.0 (0.8–5.3). The continuous pain, intermittent pain, neuropathic pain, affective descriptors, and total pain scores in the SF-MPQ-2 were 18.5 (6.5–30.0), 11.5 (0.0–28.5), 13.5 (3.0–22.3), 0.0 (0.0–10.0) and 54.0 (13.3–78.5), respectively.

**Table 3:** Self-evaluated pain intensity (N = 30).

NRS (present pain intensity)	3.0 (0.8–5.3)
SF-MPQ-2	
Continuous pain	18.5 (6.5–30.0)
Intermittent pain	11.5 (0.0–28.5)
Neuropathic pain	13.5 (3.0–22.3)
Affective descriptors	0.0 (0.0–10.0)
Total score	54.0 (13.3–78.5)

Values are presented as the median with interquartile range. The possible range in values was as follows: NRS (0–10), continuous pain (0–60), intermittent pain (0–60), neuropathic pain (0–60), affective descriptors (0–40), and total score (0–220). NRS, 10-points numerical rating scale; SF-MPQ-2, short-form McGill Pain Questionnaire 2.

### Association between pain status and marker proteins

The concentrations of the two proteins are shown in Table 2. Of 30 samples, NGF was not detected in two samples, and S100A8/A9 was not detected in eight. The medians of the standardized NGF and S100A8/A9 concentrations were 0.85 (0.32–1.70) pg/mL/cm^2^ and 0.05 (0.04–0.11) ng/mL/cm^2^, respectively.

**Fig 1:**
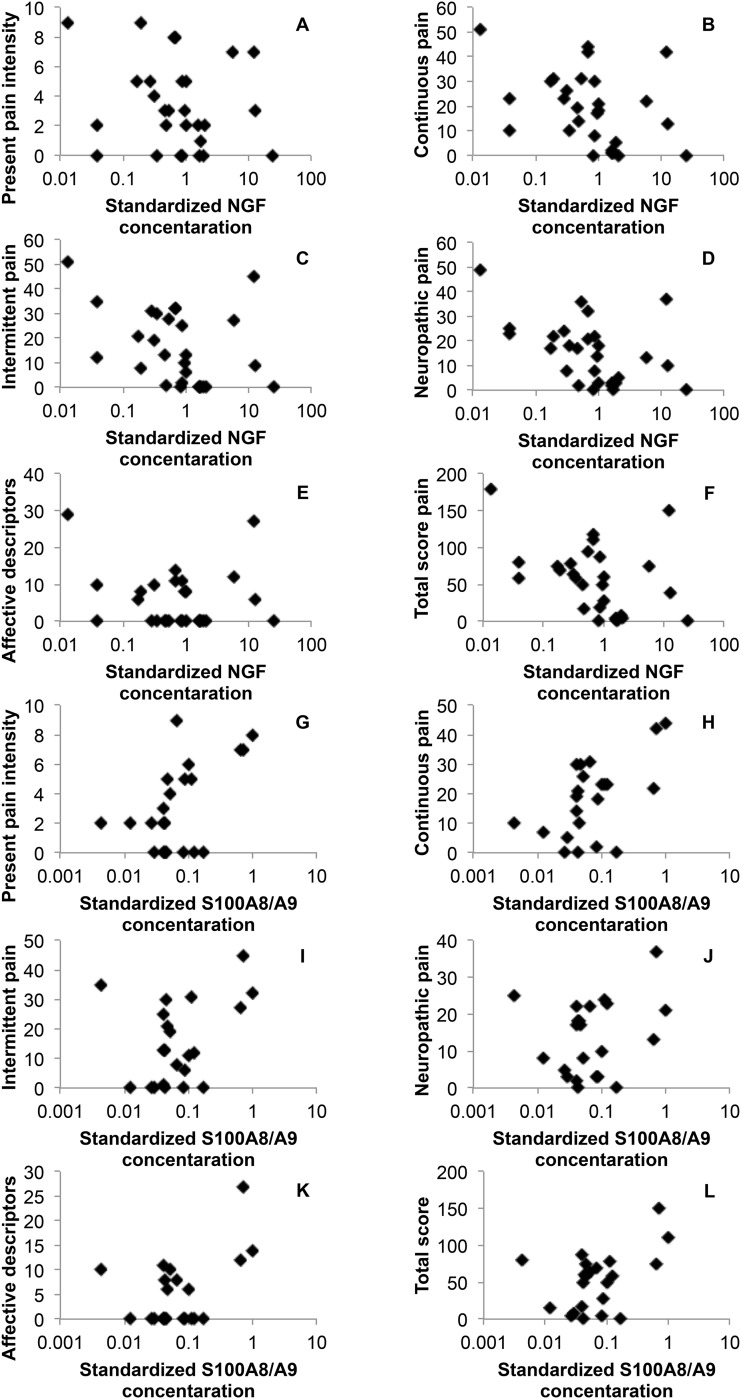
Scatter plots for the pain status and the standardized concentration of NGF or S100A8/A9. The protein concentrations were standardized according to the wound area. The horizontal axis is logarithmic axis. A–F indicate the pain status evaluated by NRS and SF-MPQ-2 and the standardized NGF concentration. G–L indicate the pain status evaluated by NRS and SF-MPQ-2 and the standardized S100A8/A9 concentration. NGF = nerve growth factor, NRS = numerical rating scale, SF-MPQ-2 = short-form McGill pain questionnaire 2.

As shown in Table 4, the standardized NGF concentration was negatively correlated with continuous pain (*ρ* = -0.47, *P* = 0.01), intermittent pain (*ρ* = -0.48, *P* = 0.01), neuropathic pain (*ρ* = -0.51, *P* = 0.01), and the total pain score (*ρ* = -0.46, *P* = 0.01). The standardized S100A8/A9 concentration was positively correlated with the present pain intensity (*ρ* = 0.46, *P* = 0.03) and continuous pain (*ρ* = 0.48, *P* = 0.03). It should be noted that wound area did not correlate with each pain intensity. The scatter plots for each pain status and each standard protein concentration are shown in Fig 1. As shown in S2 and S3 Tables, age, sex, and wound age were possibly related to standardized NGF and S100A8/A9 concentrations and pain status. Therefore, we performed stratified analyses for the three factors (S4–S6 Tables). All of the directions of correlation did not changed compared with our main results even when considering adjusting such factors except for sex. In female, a part of directions were not consistent with the main results. The standardized NGF concentration was by and large highly correlated with pain statuses in higher-age and male group even if non-significant correlations were excluded. For wound age, the standardized NGF concentration was significantly correlated with neuropathic pain in lower-wound-age group and with continuous pain in higher-wound-age group. On the other hand, the standardized S100A8/A9 concentration was significantly correlated with continuous pain in higher-age group, with NRS in male group, with NRS and continuous pain in lower-wound-age-group, and with neuropathic pain in higher-wound-age group.

**Table 4:** Associations between pain intensity and standardized NGF and S100A8/A9 concentrations.

	NGF (n = 28)		S100A8/A9 (n = 22)	
	*ρ*	*P*	*ρ*	*P*
NRS (present pain intensity)	-0.24	0.23	0.46	0.03
SF-MPQ-2				
Continuous pain	-0.47	0.01	0.48	0.03
Intermittent pain	-0.48	0.01	0.32	0.15
Neuropathic pain	-0.51	0.01	0.19	0.40
Affective descriptors	-0.11	0.58	0.30	0.18
Total score	-0.46	0.01	0.31	0.17

Values are presented as the calculated Spearman's correlation coefficient (*ρ*) followed by the *P* value. The measured protein concentrations were standardized according to the wound area. NRS, 10-points numerical rating scale; SF-MPQ-2, short-form McGill Pain Questionnaire 2; NGF, nerve growth factor.

**Fig 2:**
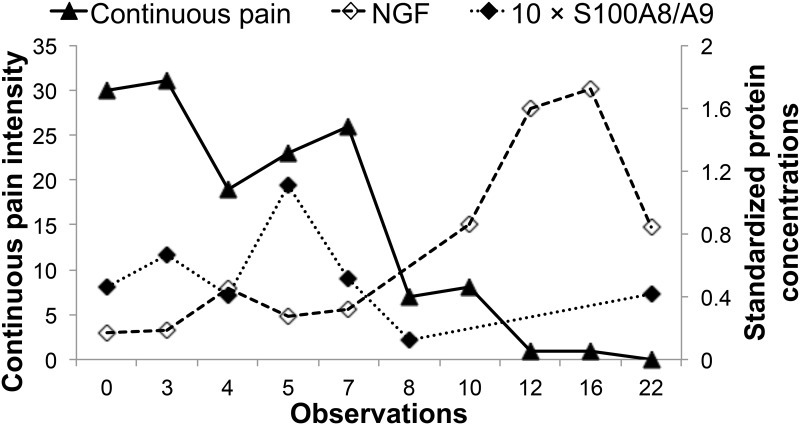
Changes in continuous pain intensity and standardized protein concentrations in ID “G”. The protein concentrations were standardized according to the wound area, and then the standardized S100A8/A9 concentration was multiplied 10-fold for clarity. The horizontal axis indicates the duration in weeks from the first examination. Numbers of NGF data were 9, and of S100A8/A9 were 7 in spite of 10 time points because NGF was not detected in one sample from observation number 8, and S100A8/A9 was not detected in samples from observation numbers 10, 12, and 16. NGF = nerve growth factor.

The changes in the continuous pain intensity and standardized protein concentrations in the ID “G” samples are shown in Fig 2. The standardized NGF concentration increased as the continuous pain intensity decreased and the standardized S100A8/A9 concentration showed comparable changes with variations in the pain intensity. The correlation coefficients in ID “G” between continuous pain intensity and the standardized protein concentrations were *ρ* = -0.86 for NGF and *ρ* = 0.64 for S100A8/A9. The correlation coefficients in all samples except the ID “G” were *ρ* = -0.39 for NGF and *ρ* = 0.52 for S100A8/A9.

## Discussion

This is the first known study showing the potential to assess wound pain intensity using VLU exudate analysis. Specifically, the study found that the standardized NGF concentration was associated with nociceptive pain, including continuous, intermittent pain, and neuropathic pain, and the standardized S100A8/A9 concentration was associated with present pain intensity and continuous pain.

Present pain intensity as evaluated by the NRS was correlated with the standardized S100A8/A9 concentration. One possible reason is the influence of NSAIDs on present pain intensity and on the S100A8/A9 expression. NSAIDs inhibit the arachidonic acid cascade that is responsible for synthesizing prostaglandin E_2_ (PGE_2_) [21]. PGE_2_ can sensitize nociceptors and has algesic action as an inflammatory mediator [27, 28]. In its extracellular function, S100A8/A9 is involved in arachidonic acid transport [20]. Moreover, previous studies demonstrated that PGE_2_ was necessary for S100A8 expression and found that prostanoid receptor antagonists inhibited the PGE_2_-mediated increase in S100A8 expression in prostate cancer cells and macrophage [29, 30]. Taken together, S100A8/A9 may accurately reflect present pain intensity.

The standardized NGF concentration was correlated with continuous, intermittent, and neuropathic pain, and the standardized S100A8/A9 concentration was correlated with continuous pain according to the SF-MPQ-2. Continuous pain is considered a nociceptive symptom due to inflammation caused by non-neural tissue damage. S100A8/A9 is also considered a damage-associated molecular pattern that induces the production of pro-inflammatory cytokines [31]. Consequently, NGF production is promoted [32]. Though many cell types produce NGF, its location associated with pain in a rat model is specific to nerve bundles [33]. A possible reason may be that extracellular free-NGF is degraded rapidly by metalloproteinase (MMP)-9 [34]. MMP-9 is abundant in VLU exudate [35, 36], and NGF itself promotes expression and activation of MMP-9 [37, 38]. NGF also activates neutrophils, which may contribute to the prolongation of inflammation in VLU and are the primary MMP-producing cell [2]. This orchestrated regulation of MMP-9 may decrease the NGF concentration in VLU exudate along with pain severity. Therefore, continuous pain showed a negative correlation with NGF and a positive correlation with S100A8/A9 in the present study. Furthermore, we confirmed the this the standardized NGF concentration was associated with inflammation of the wounds assessed by infrared thermographic assessment. This has supported the result of the present study [39].

Intermittent pain is another nociceptive symptom caused by nociceptive sensitization. The mechanical sensitization caused by NGF is well known [22, 40]. Thus, during mechanical sensitization induced by NGF, the participants may have perceived intermittent pain during mechanical stimuli such as walking or dressing change.

Neuropathic pain was correlated with the standardized NGF concentration. Thus, NGF in VLU exudate may well be an indicator of neuropathic pain. The mechanism of neuropathic pain is complex, but it is well known that NGF has important roles in neuropathic pain generation. Many studies have suggested that NGF concentration increased in neuropathic conditions, and NGF-blocking drugs effectively relieved pain when NSAIDs and opiates were ineffective [22]. Conversely, NGF may have an analgesic effect on neuropathic pain. For example, intrathecal NGF administration reduced allodynia and thermal hyperalgesia, and NGF-mimetic peptide reduced neuropathic behavior in a rat model [41, 42].

Because participants’ age, sex, and wound size were possibly confounders of the associations between two biomarkers and pain status, we performed stratified analyses based on the results indicated in S2 Table. As results, all of the directions of correlations did not changed compared with our main results even when considering adjusting such factors except for sex. Although we could not discuss the correlation coefficients with non-significance because of limited sample size, this means that at least the results of the present study were not affected by age and wound age and supports our main results indicated in Table 4. According to the results in S4 Table, the standardized NGF concentration may be used for higher-age male population as a wound pain biomarker. For the standardized S100A8/A9 concentration, any peculiarities were not found from stratified analyses. In order to test usefulness of the protein as a biomarker, we have to perform a further study with large sample size based on the present results.

Potentially, the intra-individual correlation in the ID “G” may have affected the results in the entire population because a strong correlation was observed between the pain intensity and the standardized protein concentration in the ID “G”. To test this possibility, we also calculated the correlation coefficients in all samples except the ID “G” and found that the direction of correlation was consistent with the results in the whole population. Thus, the results shown in Table 4 were not due to intra-individual correlation in the ID “G”.

The findings of the present study in which wound exudate analysis may indicate the intensity of both nociceptive and neuropathic wound pain could prove useful in establishing an objective wound pain evaluation in the future. Especially, based on these findings, we may be able to evaluate whether the source of any pain reported by a patient is wound pain or not. As a next step, we should search for more specific pain biomarkers in order to accurately distinguish between nociceptive and neuropathic pain and provide appropriate intervention in accordance with the pain quality. Furthermore, a new method of measuring the biomarker concentration is needed. Pain assessment should be performed as rapidly as possible in the clinical setting, but ELISA requires several hours to complete. Thus, development of a new detection method able to be used within several minutes is another challenge to address in subsequent studies.

The absolute values of correlation coefficient were between 0.46 and 0.51. Although the strength of the values is regarded as moderate generally, it is equal to or greater than the value of a previous study which used the skin conductance method [13]. Furthermore, our methods are superior to this method of the study because wound exudate can indicate wound pain itself specifically, whereas the skin conductance method cannot define where the painful region is.

This study has several limitations. The wound care might have affected the pain intensity assessed by SF-MPQ-2 because the scale was completed after the wound care and all of data collection doe to systems of outpatient clinics. However, the influence of wound care on pain intensity was supposed to be not so big because of following two reasons: 1. the care techniques were unified among the providers as much as possible., 2. SF-MPQ-2 asked the pain intensity during a past week not the intensity at that moment. The two biomarkers could not be detected in several samples even though we confirmed that presence of any protein was collected in all samples by measuring the total protein concentration (data not shown). A more sensitive detection method can more clearly reveal the associations between the pain status and the biomarker concentrations. Moreover, it must be careful to generalize our findings because of limited sample size. Potentially, small sample size may cause type II error. It was possibly occurred that we failed to reject the null hypothesis when it was false. As shown in supporting data, we confirmed that almost all of the directions of correlation did not changed compared with our main results even when considering adjusting such factors. In parts of the results, however, we could not evaluate the results precisely because of limited statistical power. We would like to perform a study which has high statistical power as a next step. Additionally, our findings may be difficult to generalize to patients with VLU accompanied with systemic infection because the patients with severe state of inflammation/infection evaluated by DESIGN tool did not participate. A previous study demonstrated that bacteria directly activate nociceptors, and that the inflammatory response is not necessary for bacteria-induced pain [43] which may have other pathways for generation.

As a conclusion, this study found that the NGF and S100A8/A9 in VLU exudate were associated with the self-reported pain status. Thus, these two proteins may be useful for an objective pain evaluation. Further studies such as a proteome analysis using an animal model are needed to identify more specific biomarkers for nociceptive and neuropathic pain. Furthermore, the usefulness of these various biomarkers should be validated in patients with cognitive impairment.

## Supporting Information

S1 Table Shapiro-Wilk test for confirming normal distribution. NGF, nerve growth factor; NRS, 10-points numerical rating scale; SF-MPQ-2, short-form McGill Pain Questionnaire 2.
(DOCX)

S2 Table Associations between participant characteristics and marker concentrations. Values are presented as the median with interquartile range. Each value of two groups was compared using Wilcoxon’s rank sum test. NGF, nerve growth factor.
(DOCX)

S3 Table Associations between participant characteristics and pain intensities. Values are presented as the median with interquartile range. Each value of two groups was compared using Wilcoxon’s rank sum test. NRS, 10-points numerical rating scale; SF-MPQ-2, short-form McGill Pain Questionnaire 2.
(DOCX)

S4 Table Stratified analysis by age for association between pain intensity and standardized NGF and S100A8/A9. Values are presented as the calculated Spearman's correlation coefficient (*ρ*) followed by the *P* value. The measured protein concentrations were standardized according to the wound area. NRS, 10-points numerical rating scale; SF-MPQ-2, short-form McGill Pain Questionnaire 2; NGF, nerve growth factor.
(DOCX)

S5 Table Stratified analysis by sex for association between pain intensity and standardized NGF and S100A8/A9. Values are presented as the calculated Spearman's correlation coefficient (*ρ*) followed by the *P* value. The measured protein concentrations were standardized according to the wound area. NRS, 10-points numerical rating scale; SF-MPQ-2, short-form McGill Pain Questionnaire 2; NGF, nerve growth factor.
(DOCX)

S6 Table Stratified analysis by wound age for association between pain intensity and standardized NGF and S100A8/A9. Values are presented as the calculated Spearman's correlation coefficient (*ρ*) followed by the *P* value. The measured protein concentrations were standardized according to the wound area. NRS, 10-points numerical rating scale; SF-MPQ-2, short-form McGill Pain Questionnaire 2; NGF, nerve growth factor.
(DOCX)
