# Supplementary material for: Can Wound Exudate from Venous Leg Ulcers Measure Wound Pain Status?: A Pilot Study
Source: PLoS One. 2016 Dec 9;11(12):e0167478. doi: 10.1371/journal.pone.0167478 (PMC5147907; doi:10.1371/journal.pone.0167478)
Supplement: S1 Table — NGF, nerve growth factor; NRS, 10-points numerical rating scale; SF-MPQ-2, short-form McGill Pain Questionnaire 2. (DOCX) [file pone.0167478.s001.docx]

| **S1 Table.** Shapiro-Wilk test for confirming normal distribution | | | |
| --- | --- | --- | --- |
|  | *N* | *W* | *P* |
| Age | 30 | 0.81 | 0.00 |
| BMI | 30 | 0.83 | 0.00 |
| Wound age | 25 | 0.94 | 0.15 |
| Wound area | 30 | 0.87 | 0.00 |
| NGF | 30 | 0.97 | 0.54 |
| S100A8A9 | 30 | 0.59 | 0.00 |
| NRS | 30 | 0.94 | 0.09 |
| SF-MPQ-2 |  |  |  |
| Continuous pain | 30 | 0.94 | 0.11 |
| Intermittent pain | 30 | 0.90 | 0.01 |
| Neuropathic pain | 30 | 0.92 | 0.03 |
| Affective descriptors | 30 | 0.81 | 0.00 |
| Total score | 30 | 0.92 | 0.03 |
| NGF, nerve growth factor; NRS, 10-points numerical rating scale; SF-MPQ-2, short-form McGill Pain Questionnaire 2. | | | |
