# Supplementary material for: Can Wound Exudate from Venous Leg Ulcers Measure Wound Pain Status?: A Pilot Study
Source: PLoS One. 2016 Dec 9;11(12):e0167478. doi: 10.1371/journal.pone.0167478 (PMC5147907; doi:10.1371/journal.pone.0167478)
Supplement: S2 Table — Values are presented as the median with interquartile range. Each value of two groups was compared using Wilcoxon’s rank sum test. NGF, nerve growth factor. (DOCX) [file pone.0167478.s002.docx]

| **S2 Table.** Associations between participant characteristics and marker concentrations | | | | | | | |
| --- | --- | --- | --- | --- | --- | --- | --- |
|  | Standardized NGF concentration | | |  | Standardized S100A8/A9 concentration | | |
|  | pg/mL/cm2 | *z* | *P* |  | ng/mL/cm2 | *z* | *P* |
| Age |  | 2.48 | 0.01 |  |  | 1.54 | 0.12 |
| < 76.5 | 1.00 (0.68−5.73) |  |  |  | 0.09 (0.04−0.65) |  |  |
| ≥ 76.5 | 0.40 (0.19−0.86) |  |  |  | 0.05 (0.03−0.07) |  |  |
| Sex |  | -0.32 | 0.75 |  |  | 0.82 | 0.41 |
| Male | 0.84 (0.32−1.60) |  |  |  | 0.06 (0.04−0.11) |  |  |
| Female | 1.00 (0.34−1.92) |  |  |  | 0.04 (0.03−0.10) |  |  |
| Wound age |  | 0.98 | 0.33 |  |  | 1.78 | 0.08 |
| < 9 | 0.98 (0.50−3.82) |  |  |  | 0.10 (0.05−0.65) |  |  |
| ≥ 9 | 0.69 (0.30−1.30) |  |  |  | 0.04 (0.04−0.06) |  |  |
| Values are presented as the median with interquartile range. Each value of two groups was compared using Wilcoxon’s rank sum test. NGF, nerve growth factor | | | | | | | |
