# Supplementary material for: Can Wound Exudate from Venous Leg Ulcers Measure Wound Pain Status?: A Pilot Study
Source: PLoS One. 2016 Dec 9;11(12):e0167478. doi: 10.1371/journal.pone.0167478 (PMC5147907; doi:10.1371/journal.pone.0167478)
Supplement: S3 Table — Values are presented as the median with interquartile range. Each value of two groups was compared using Wilcoxon’s rank sum test. NRS, 10-points numerical rating scale; SF-MPQ-2, short-form McGill Pain Questionnaire 2. (DOCX) [file pone.0167478.s003.docx]

| **S3 Table.** Associations between participant characteristics and pain intensities | | | | | | | | | | | | | | | | | | | | | | | |
| --- | --- | --- | --- | --- | --- | --- | --- | --- | --- | --- | --- | --- | --- | --- | --- | --- | --- | --- | --- | --- | --- | --- | --- |
|  | NRS | | |  | SM-MPQ-2 | | | | | | | | | | | | | | | | | | |
|  |  |  |  |  | Continuous pain | | |  | Intermittent pain | | |  | Neuropathic pain | | |  | Affective descriptors | | |  | Total score | | |
|  | Point | *z* | *P* |  | Point | *z* | *P* |  | Point | *z* | *P* |  | Point | *z* | *P* |  | Point | *z* | *P* |  | Point | *z* | *P* |
| Age |  | 1.18 | 0.24 |  |  | 1.00 | 0.32 |  |  | 0.65 | 0.52 |  |  | 0.17 | 0.87 |  |  | 2.39 | 0.02 |  |  | 0.79 | 0.43 |
| < 76.5 | 3.0 (2.0−7.0) |  |  |  | 21.0 (13.0−42.0) |  |  |  | 11.0 (1.0−32.0) |  |  |  | 13.0 (3.0−22.0) |  |  |  | 8.0 (0.0−12.0) |  |  |  | 50.0 (17.0−111.0) |  |  |
| ≥ 76.5 | 2.0 (0.0−5.0) |  |  |  | 10.0 (5.0−26.0) |  |  |  | 12.0 (0.0−28.0) |  |  |  | 17.0 (3.0−23.0) |  |  |  | 0.0 (0.0−6.0) |  |  |  | 58.0 (8.0−74.0) |  |  |
| Sex |  | 2.16 | 0.03 |  |  | 0.61 | 0.54 |  |  | -0.53 | 0.60 |  |  | -1.34 | 0.18 |  |  | 0.74 | 0.46 |  |  | -0.39 | 0.70 |
| Male | 4.0 (2.0−6.0) |  |  |  | 19.0 (8.0−30.0) |  |  |  | 10.0 (1.0−25.0) |  |  |  | 10.0 (3.0−21.0) |  |  |  | 6.0 (0.0−10.0) |  |  |  | 49.0 (17.0−74.0) |  |  |
| Female | 2.0 (0.0−2.0) |  |  |  | 10.0 (5.0−23.0) |  |  |  | 13.0 (0.0−30.0) |  |  |  | 18.0 (5.0−25.0) |  |  |  | 0.0 (0.0−8.0) |  |  |  | 58.0 (8.0−70.0) |  |  |
| Wound age |  | 1.00 | 0.32 |  |  | 0.65 | 0.52 |  |  | 1.61 | 0.11 |  |  | 0.78 | 0.44 |  |  | 1.96 | 0.05 |  |  | 1.17 | 0.24 |
| < 9 | 5.0 (0.0−7.0) |  |  |  | 18.0 (10.0−42.0) |  |  |  | 27.0 (6.0−32.0) |  |  |  | 14.0 (3.0−25.0) |  |  |  | 8.0 (0.0−12.0) |  |  |  | 58.0 (27.0−111.0) |  |  |
| ≥ 9 | 2.0 (2.0−7.0) |  |  |  | 10.0 (7.0−26.0) |  |  |  | 9.0 (0.0−19.0) |  |  |  | 10.0 (5.0−22.0) |  |  |  | 0.0 (0.0−6.0) |  |  |  | 49.0 (15.0−69.0) |  |  |
| Values are presented as the median with interquartile range. Each value of two groups was compared using Wilcoxon’s rank sum test.  NRS, 10-points numerical rating scale; SF-MPQ-2, short-form McGill Pain Questionnaire 2. | | | | | | | | | | | | | | | | | | | | | | | |
