# Supplementary material for: Can Wound Exudate from Venous Leg Ulcers Measure Wound Pain Status?: A Pilot Study
Source: PLoS One. 2016 Dec 9;11(12):e0167478. doi: 10.1371/journal.pone.0167478 (PMC5147907; doi:10.1371/journal.pone.0167478)
Supplement: S4 Table — Values are presented as the calculated Spearman's correlation coefficient (ρ) followed by the P value. The measured protein concentrations were standardized according to the wound area. NRS, 10-points numerical rating scale; SF-MPQ-2, short-form McGill Pain Questionnaire 2; NGF, nerve growth factor. (DOCX) [file pone.0167478.s004.docx]

| **S4 Table.** Stratified analysis by age for association between pain intensity and standardized NGF and S100A8/A9 | | | | | | | | | | | |
| --- | --- | --- | --- | --- | --- | --- | --- | --- | --- | --- | --- |
|  | Standardized NGF concentration | | | | |  | Standardized S100A8/A9 concentrations | | | | |
|  | Age < 76.5 | |  | Age ≥ 76.5 | |  | Age < 76.5 | |  | Age ≥ 76.5 | |
|  | *ρ* | *P* |  | *ρ* | *P* |  | *ρ* | *P* |  | *ρ* | *P* |
| NRS | -0.37 | 0.19 |  | -0.28 | 0.33 |  | 0.68 | 0.02 |  | 0.33 | 0.32 |
| SF-MPQ-2 |  |  |  |  |  |  |  |  |  |  |  |
| Continuous pain | -0.56 | 0.04 |  | -0.65 | 0.01 |  | 0.51 | 0.11 |  | 0.68 | 0.02 |
| Intermittent pain | -0.39 | 0.16 |  | -0.69 | 0.01 |  | 0.56 | 0.07 |  | 0.15 | 0.67 |
| Neuropathic pain | -0.39 | 0.17 |  | -0.70 | 0.01 |  | 0.25 | 0.46 |  | 0.32 | 0.35 |
| Affective descriptors | -0.29 | 0.32 |  | -0.57 | 0.03 |  | 0.57 | 0.07 |  | -0.01 | 0.99 |
| Total score | -0.44 | 0.12 |  | -0.69 | 0.01 |  | 0.45 | 0.17 |  | 0.30 | 0.38 |
| Values are presented as the calculated Spearman's correlation coefficient (*ρ*) followed by the *P* value. The measured protein concentrations were standardized according to the wound area. NRS, 10-points numerical rating scale; SF-MPQ-2, short-form McGill Pain Questionnaire 2; NGF, nerve growth factor. | | | | | | | | | | | |
