# Supplementary material for: Can Wound Exudate from Venous Leg Ulcers Measure Wound Pain Status?: A Pilot Study
Source: PLoS One. 2016 Dec 9;11(12):e0167478. doi: 10.1371/journal.pone.0167478 (PMC5147907; doi:10.1371/journal.pone.0167478)
Supplement: S5 Table — Values are presented as the calculated Spearman's correlation coefficient (ρ) followed by the P value. The measured protein concentrations were standardized according to the wound area. NRS, 10-points numerical rating scale; SF-MPQ-2, short-form McGill Pain Questionnaire 2; NGF, nerve growth factor. (DOCX) [file pone.0167478.s005.docx]

| **S5 Table.** Stratified analysis by sex for association between pain intensity and standardized NGF and S100A8/A9 | | | | | | | | | | | |
| --- | --- | --- | --- | --- | --- | --- | --- | --- | --- | --- | --- |
|  | Standardized NGF concentration | | | | |  | Standardized S100A8/A9 concentrations | | | | |
|  | Male | |  | Female | |  | Male | |  | Female | |
|  | ρ | P |  | ρ | P |  | ρ | P |  | ρ | P |
| NRS | -0.51 | 0.02 |  | 0.36 | 0.34 |  | 0.60 | 0.02 |  | -0.09 | 0.83 |
| SF-MPQ-2 |  |  |  |  |  |  |  |  |  |  |  |
| Continuous pain | -0.66 | 0.00 |  | -0.19 | 0.62 |  | 0.24 | 0.42 |  | 0.60 | 0.12 |
| Intermittent pain | -0.49 | 0.03 |  | -0.20 | 0.60 |  | 0.42 | 0.13 |  | 0.22 | 0.60 |
| Neuropathic pain | -0.60 | 0.01 |  | -0.21 | 0.59 |  | 0.13 | 0.66 |  | 0.27 | 0.53 |
| Affective descriptors | -0.24 | 0.32 |  | 0.17 | 0.67 |  | 0.35 | 0.22 |  | 0.05 | 0.90 |
| Total score | -0.54 | 0.02 |  | -0.13 | 0.73 |  | 0.32 | 0.26 |  | 0.19 | 0.65 |
| Values are presented as the calculated Spearman's correlation coefficient (*ρ*) followed by the *P* value. The measured protein concentrations were standardized according to the wound area. NRS, 10-points numerical rating scale; SF-MPQ-2, short-form McGill Pain Questionnaire 2; NGF, nerve growth factor. | | | | | | | | | | | |
