# Supplementary material for: Can Wound Exudate from Venous Leg Ulcers Measure Wound Pain Status?: A Pilot Study
Source: PLoS One. 2016 Dec 9;11(12):e0167478. doi: 10.1371/journal.pone.0167478 (PMC5147907; doi:10.1371/journal.pone.0167478)
Supplement: S6 Table — Values are presented as the calculated Spearman's correlation coefficient (ρ) followed by the P value. The measured protein concentrations were standardized according to the wound area. NRS, 10-points numerical rating scale; SF-MPQ-2, short-form McGill Pain Questionnaire 2; NGF, nerve growth factor. (DOCX) [file pone.0167478.s006.docx]

| **S6 Table.** Stratified analysis by wound age for association between pain intensity and standardized NGF and S100A8/A9 | | | | | | | | | | | |
| --- | --- | --- | --- | --- | --- | --- | --- | --- | --- | --- | --- |
|  | Standardized NGF concentration | | | | |  | Standardized S100A8/A9 concentrations | | | | |
|  | Wound age < 9 | |  | Wound age ≥ 9 | |  | Wound age < 9 | |  | Wound age ≥ 9 | |
|  | ρ | P |  | ρ | P |  | ρ | P |  | ρ | P |
| NRS | -0.34 | 0.29 |  | -0.36 | 0.17 |  | 0.74 | 0.01 |  | 0.38 | 0.23 |
| SF-MPQ-2 |  |  |  |  |  |  |  |  |  |  |  |
| Continuous pain | -0.39 | 0.22 |  | -0.64 | 0.01 |  | 0.63 | 0.05 |  | 0.56 | 0.06 |
| Intermittent pain | -0.57 | 0.05 |  | -0.48 | 0.06 |  | 0.26 | 0.46 |  | 0.46 | 0.13 |
| Neuropathic pain | -0.60 | 0.04 |  | -0.49 | 0.05 |  | 0.19 | 0.60 |  | 0.60 | 0.04 |
| Affective descriptors | -0.25 | 0.43 |  | -0.06 | 0.83 |  | 0.61 | 0.06 |  | 0.14 | 0.67 |
| Total score | -0.50 | 0.10 |  | -0.50 | 0.05 |  | 0.37 | 0.29 |  | 0.47 | 0.12 |
| Values are presented as the calculated Spearman's correlation coefficient (*ρ*) followed by the *P* value. The measured protein concentrations were standardized according to the wound area. NRS, 10-points numerical rating scale; SF-MPQ-2, short-form McGill Pain Questionnaire 2; NGF, nerve growth factor. | | | | | | | | | | | |
